# Flour nutritional profile, and soxhlet-extracted oil physicochemical breakdown-storage performance of white melon (*Cucumeropsis mannii* Naudin) seed varieties from Southeast Nigeria

**DOI:** 10.1371/journal.pone.0282974

**Published:** 2023-05-11

**Authors:** Solomon I. Nwoke, Queency N. Okechukwu, Fabian U. Ugwuona, Moses Ojukwu, Hanna Skendrović, Szymon Juchniewicaz, Katarzyna Leicht, Charles Odilichukwu R. Okpala, Małgorzata Korzeniowska

**Affiliations:** 1 Department of Food Science and Technology, Michael Okpara University of Agriculture, Umudike, Abia State, Nigeria; 2 Institute of Chemical Technology, Ural Federal University, Named After the First President of Russia BN, Yeltsin, Yekaterinburg, Russia; 3 Department of Food Science and Technology, School of Engineering and Engineering Technology, Federal University of Technology, Owerri, Imo State, Nigeria; 4 Faculty of Food Technology and Biotechnology, University of Zagreb, Zagreb, Croatia; 5 Department of Functional Food Products Development, Wrocław University of Environmental and Life Sciences, Wrocław, Poland; 6 UGA Cooperative Extension, College of Agricultural and Environmental Sciences, University of Georgia Athens, Athens, GA, United States of America; Botswana International University of Science and Technology (BIUST), BOTSWANA

## Abstract

White melon (*Cucumeropsis mannii* Naudin), is among common and yet underutilized oil seed crop within the West African region, does not have sufficient information specific to its nutrient composition for foreign consumers. To supplement existing information, therefore, we investigated the nutritional profile of defatted and full-fat flour, alongside physicochemical breakdown and storage performance of soxhlet-extracted oil from two white melon (*C*. *mannii*) seed varieties found in Southeast Nigeria. Nutritional profile involved the determinations of proximate composition, minerals, vitamins, functional properties as well as amino acid profile. Physicochemical breakdown involved the determinations of fatty acid profile, lipid breakdown parameters, as well as associated physical attributes. Results showed defatting of flours increased the protein (69.04%), carbohydrates (16.26%), crude fiber (2.68%), ash (11.9%), mineral (Na ranging from 223.92–246.99 mg/100g), and vitamin contents (Vit B_1_ ranging from 0.453–0.712 mg/100g). Total amino acid differed slightly when comparing miniature (30.36 g/100g) and large (22.36 g/100g) seeds. Soxhlet-extracted oil possessed low thiobarbituric acid, acid, and peroxide values (0.030 and 0.038 mg MDA/kg, 1.08 and 1.27 mg KOH/g, and 2.95 and 3,94 mEqO2/kg, for large and miniature seeds respectively), and peak linoleic acid (5 and 6.45 mg/ml, for miniature and large seeds respectively). During storage, the thiobarbituric acid and peroxide values of soxhlet-extracted oil increased yet within acceptable limits.

## Introduction

Food processing in Nigeria, and extending to the West Africa sub-region has evolved over the decades. Applicable to plant-based products, food processing range from small to large-scale operations [1]. Food processing, depending on the product type, would typically involve major stages like receiving the fresh product, washing to remove dirt or debris from the field, cutting or slicing, and drying/milling to form the flour before packaging/storage [1, 2]. There is paucity of both finished and processed foods that are of appropriate nutritional quality especially those that meet the needs of developing nations’ growing population. More so, the challenges of food security continue to persist [2] which is why neglected, and underutilized food crops require further product development strategies [2, 3]. Besides, successful food product creation/innovation requires understanding the nutritional, functional, and storage aspects of the (food) crop which serves as the raw material [3].

Traditional African vegetables and oil crops are considered with capacity to generate revenue and provide food security, but their industrial potential tends to be undervalued. The merits of such food crops include its indigenous nature, high adaptability to the local climate, and requirement of low cultivation inputs [3, 4]. Amongst such underutilized and undervalued African vegetables that serve as a cheap nutrient is the white melon (*Cucumeropsis mannii* Naudin*)*, an important leguminous crop widely grown and consumed in western (Benin, Nigeria, Côte d’Ivoire, and Ghana) and central African (Cameroon, Republic Democratic of Congo) sub-region [4]. It is a member of the *Cucurbitaceae* family, which includes groups of cultivars that produce seeds that have a juicy pale yellow or green flesh that tastes bitter and is commonly mistaken for *Citrullus spp*. (lanatus, *mucosospermus*, *Vulgaris or colocynthis* [5–7]. A tendril climber, white melon is a typically annual creeping herb with hairy stems and a lobed leaf plant with a fibrous and shallow root system that thrives throughout the Savannah belts of Nigeria as a subsidiary crop [8]. The seeds of *C*. *mannii*, popularly called ‘egusi’ in southeast Nigeria, are protein-rich with high (50%) edible oil, competing with peanuts, fluted pumpkin, and soybean [9]. The seed oil is highly polyunsaturated, having about 63% linoleic acid and 16% oleic acid, and is also a rich source of vitamins C and B2, minerals, riboflavin, fats, and carbohydrate [4, 9]. Particularly integral in soup making and other traditional dishes across Nigeria, the ’egusi’ product relishes high sensory and nutritional qualities [2, 5]. Seeds of *C*. *mannii* are not limited to food and medicinal purposes alone [10], but are also relevant in cosmetics [11], serving also as a potential biodiesel feedstock [7, 12].

Despite the available information regarding its nutritional potential, the production of *C*. *mannii* species in Africa appears to be declining [4], limiting further research into its use in food processing. Furthermore, earlier researchers understood that the studies on *Cucurbitaceae spp* appear to be limited to *C*. *colocynthis*, *C vulgaris*, and *Telfairia occidentalis* [2, 4]. Moreover, the quality of food products would be strongly dependent on nutritional information. Even though *C*. *mannii* is a common food in Nigeria and extends to the West African region, the nutritional information appears not readily available, especially for foreign consumption. Further, there is paucity of relevant information about the flour quality characteristics of *C*. *mannii* seeds and its derived oil as processed in Southeast Nigeria. Harnessing such information of the melon (*C*. *mannii*) crop/seed from different varieties would help prove the product development importance, and increase its food/industrial applications. Moreover, understanding the nutritional, functional, physicochemical, and storage aspects of white melon (*C*. *mannii*) seed flour and oils would pave a way for further innovative food product creation, dietary supplements, and/or as nutraceuticals in treatments of medical value. In this context, therefore, this current work investigated the flour nutritional profile and Soxhlet-extracted oil physicochemical breakdown-storage performance of melon (*C*. *mannii*) seed varieties in Southeast Nigeria.

## Materials and methods

### Schematic overview of the experimental program

**Fig 1:**
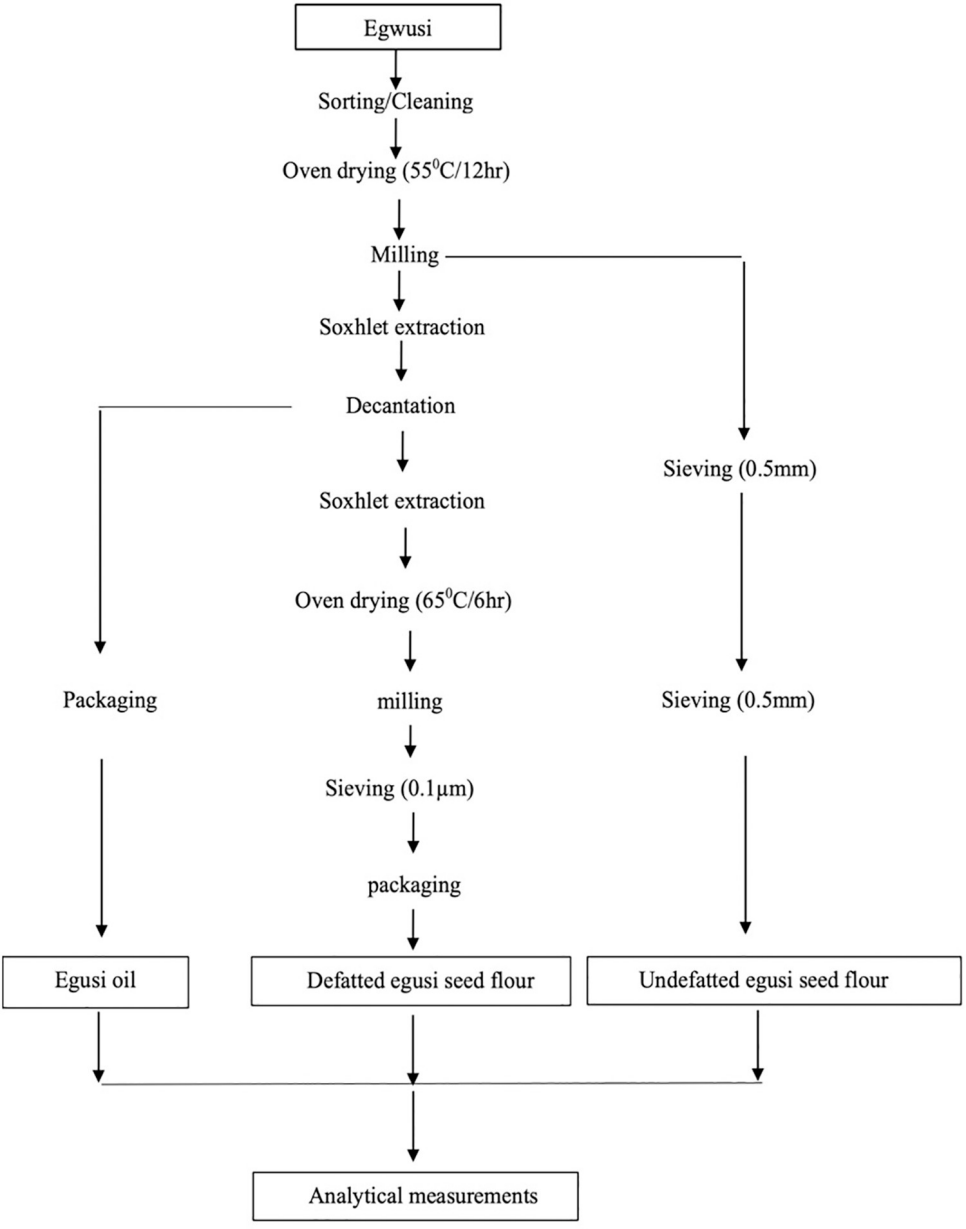
The schematic overview of the experimental program, with major steps, from the preparation of the egusi (white melon seeds), through the milling stage and subsequent sieving to the extraction of (egusi) full-fat flour through decantation, to the extraction of the (egusi) oil through Soxhlet extraction and subsequent stages to derive the defatted egusi seed flour, followed by their analytical measurements.

Fig 1 shows the schematic overview of the experimental program, with major steps, from the preparation of the egusi (white melon seeds), through the milling stage and subsequent sieving to obtain (egusi) full-fat flour through decantation, to the extraction of the (egusi) oil through Soxhlet extraction and subsequent stages to derive the defatted egusi seed flour, followed by their analytical measurements. For emphasis, this work has been directed to find out more about the nutritional profile of (*C*. *mannii*) seeds from South-East Nigeria by comparing the varieties of defatted and full-fat melon flour gotten from them, and the physicochemical and storage attributes of the extracted oils. All chemicals and reagents used were of an analytical grade standard. Furthermore, all analytical measurements conducted adhered to the relevant guidelines proved by Michael Okpara University of Agriculture, Umudike, Abia State, as well as the International Institute of Tropical Agriculture (IITA), Ibadan, Oyo State, Nigeria.

### Collection of plant materials

**Fig 2:**
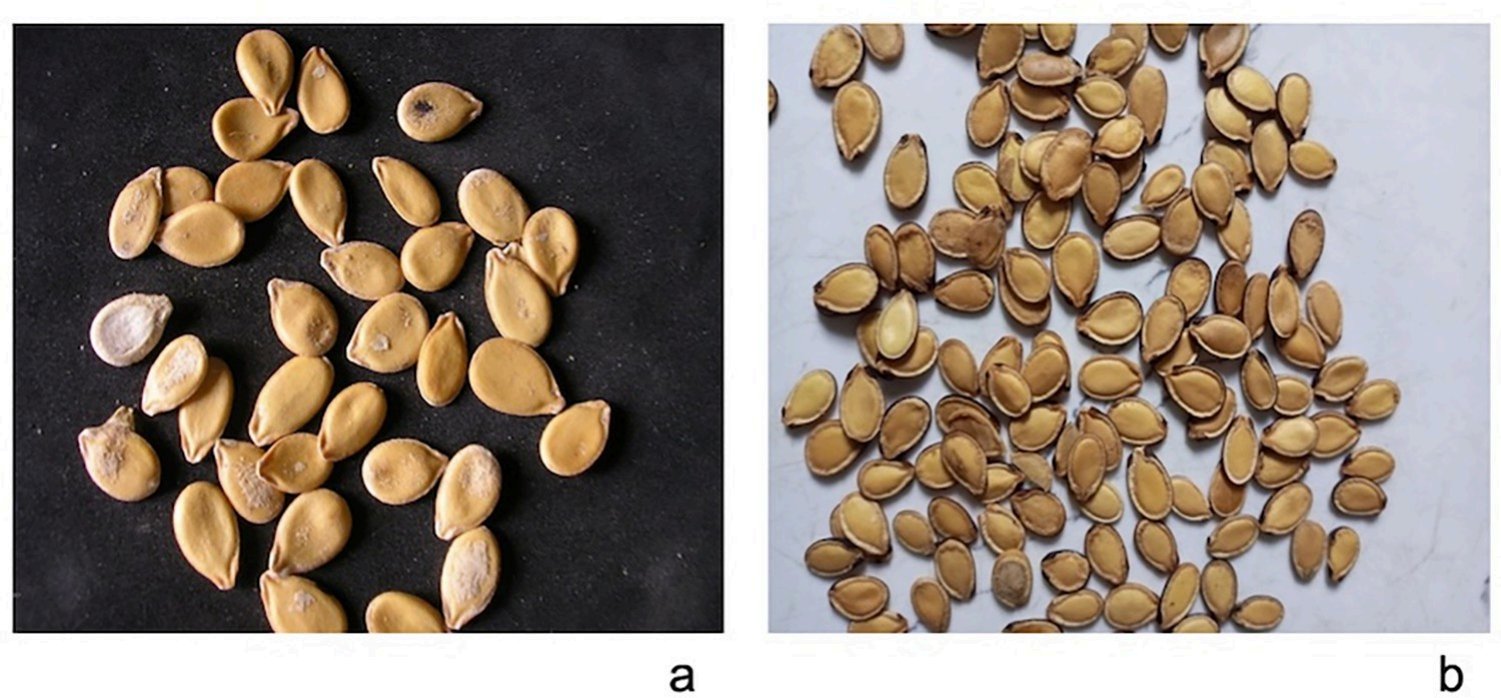
Unshelled “Serewe" type of egusi (a) miniature (b) large.

The two local varieties of melon (*C*. *mannii*), namely “Serewe” miniature, and “Serewe” large (both with no seed edge, where the miniature seed being a morpho-type of the large one) were bought at Ogige market in Nsukka, Enugu State, Nigeria. Furthermore, Fig 2 shows the photo images of “Serewe” miniature, seeds (Fig 2A) and “Serewe” large seeds (Fig 2B). The authentication and validation of the samples were performed by Mr. Felix Nwafor at the Faculty of Pharmaceutical Sciences, University of Nigeria, Nsukka, Nigeria. The samples were then taken to the Department of Food Science and Technology of Michael Okpara University of Agriculture, Umudike, Abia State, for further processing to produce flour and oil.

### Preparation of melon seed flour and Soxhlet oil extraction

As shown in Fig 1, the oil has to be produced from defatted and full-fat flours, which had come from the studied melon seeds. In a greater detail, the preparation of melon seed oil required major stages from the assembly of egusi seeds, through Soxhlet extraction, up to its production, that is, the egusi oil, as well as the defatted /full-fat [egusi seed] flour. After sorting and cleaning, each seed variety was divided into two equal portions, oven dried for 12 h at 55°C and subsequently milled to obtain the flour powder. One part was sieved through a 0.5 mm sieve and packaged in airtight containers for analysis. The other part was divided into two, where one part was soaked in petroleum ether mixture in a Soxhlet apparatus for defatting/oil extraction and decantation after 15 h to separate the oil from the melon residue and oven-dried at 65°C for 6 h according to AOAC [13]. Whilst the extracted oil was packaged, the dried flour was further milled to finer particles (to provide a miniature size) and then sieved with a 0.1 μm mesh to obtain flour of fine texture. The defatted melon seed flour was packaged in airtight containers for analysis. Whilst the flour and oil analyses were performed at the Department of Food Science and Technology, Michael Okpara University of Agriculture, Umudike, Abia State, both amino and fatty acid profiles were carried out at the International Institute of Tropical Agriculture (IITA), Ibadan, Oyo State, Nigeria.

### Analytical measurements

#### Nutritional profile of *C*. *mannii* flours

*a) Determination of proximate composition*. All flour samples were analyzed for the proximate composition according to AOAC [13]. Specifically, the evaporation method was used to determine the moisture content, whereas the gravimetric method was used to determine the crude fiber content. Additionally, ash content was determined by way of muffle furnace (model no: SX2-2.5–12, China) at temperatures of 550–650°C. Crude protein was conducted using the Kjeldahl method, and the total nitrogen content was calculated by a factor of 6.25. The continuous solvent extraction method was used to determine the fat content. More so, the carbohydrate content was estimated by difference, as depicted by the Eq 1 below:

%Carbohydrate=100–(%Moisture+%Ash+%Protein+%Fat+%CrudeFiber). 1

*b) Determination of minerals*, *vitamins*, *and carotenoids*. To determine the mineral components, the ash content already captured from flour samples was used. Specifically, calcium (Ca) and magnesium (Mg) were quantified using the EDTA complexometric titration method [14], whereas the phosphorus (P) were colorimetrically quantified using the phosphomolybdate method [14]. Furthermore, potassium (K) and sodium (Na) were conducted by flame photometry (Corning, UK, Model 405) [13]. In addition, the iron and zinc content was conducted by wet digestion [13].

Vitamins B_1_ (thiamine), B_2_ (riboflavin), and B_3_ (niacin) were conducted spectrophotometrically using a Jenway electronic spectrophotometer (model no: 7305; England) [13]. Total carotenoids were conducted as described by de Carvalho et al [15] with slight modifications. Carotenoids were extracted by mixing 3 g of the sample with 50 mL of cold acetone. The residue was filtered in a Buchner funnel equipped with Whatman paper and repeated until the extraction was colorless. The total extract was transferred to a separate funnel (250 mL) having 20 mL of petroleum ether. Distilled water (1 L) was used to wash the organic phase and the separated aqueous phase was discarded. This procedure was repeated four times until no residual solvent remained and the extract was transferred to a 50 mL volumetric flask having 15 g of anhydrous sodium sulfate. The volume was made up of petroleum ether, and the samples were read at 450 nm. The total carotenoid content was calculated using the Eq 2 as below:

$$Carotenoid\;content(\mu g/g)=\frac{A*V(ml)*10^{4}}{A_{1cm}^{1\%}*P(g)}$$ 2

where A = Absorbance; V = Total extract volume; P = sample weight; $A_{1cm}^{1\%}$ = 2592 (β-carotene Extinction Coefficient in petroleum ether). The result was multiplied by one hundred to give the carotenoid content in μg/100g

*c) Determination of functional properties*. Oil and water absorption (OAC and WAC), emulsion capacity (EC), foam capacity (FC), bulk density (BD), and swelling index (SI) were conducted following previously described methods [13, 16] with slight modifications. For OAC and WAC, each sample was individually weighed at 1 g and placed in a fresh, dry centrifuge tube. The samples were combined with distilled water or oil up to a 10 mL dispersion, properly mixed for 30 s, and then left to stand for 5 min at room temperature before being centrifuged for 5 min at 1000 rpm. Directly from the graduated centrifuge tube, the amount of free water or oil (supernatant) was measured. WAC and OAC were examined as mL water bound per gram of flour. For EC, 25 mL of distilled water and 1.8 g of flour samples were blended at the highest speed using a Moulinex-Optiblend 2000 (Trio, China) blender. 12.5 mL of vegetable oil was added, and 1 min of high-speed mixing followed. The created emulsion was divided into two 12 mL centrifuge tubes and spun at 5200 rpm for 5 min in a centrifuge (225, Fisher Scientific, Pittsburg, PA, USA). The ratio of the height of the emulsion layer to the total height of the mixture was calculated as emulsion capacity in percentage. For FC, about 2 g of samples were weighed for each and blended with 100ml of distilled water using a Moulinex-Optiblend 2000 (Trio, China) blender and the suspension whipped at 1600 rpm for 5 min, and thereafter recorded after 30 sec. Foam capacity is expressed as a percent increase in volume after whipping. For SI, 1 g of flour and 10 mL of distilled water were heated for 30 minutes at 80°C while being constantly shaken in a tube and then centrifuged at 1000 g for 15 min. The weight of the paste after decanting the supernatant was taken. Swelling index was calculated by dividing the weight of the paste by the weight of the flour. For BD, samples were added to a graduated measuring cylinder with a 10 mL capacity, and the first volume was noted. To remove air and create a vacuum, the cylinder was repeatedly tapped on the side until the volume was constant. After it leveled, the final volume was recorded, and the samples’ bulk density (g/mL) was computed using the samples’ weight and volume.
*d) Determination of amino acid profile*. The amino acid profile of the defatted flour samples was conducted using the ion exchange chromatography-based PTH Amino Acid Analyzer (Applied Biosystems, Model 120A, Massachusetts, USA) as described by Ibegbulem et al. [17] with slight modifications. About 500 mg of the defatted sample was transferred into a glass ampoule. Then, 7 mL of 6 N HCl was added, and oxygen was expelled by passing nitrogen into the ampoule. The glass ampoule was then sealed with the Bunsen burner flame and placed in an oven (Gallenkamp, Oven 300 plus series, England) preheated at 105°C ± 5°C for 22 hrs. The ampoule was cooled before it was broken open at the tip and its contents were filtered using Whatman No 52-filter paper to remove the hummus. The filtrate was then evaporated to dryness using the rotary evaporator (Büchi Rotavapor R-200, Büchi, Flawil, Switzerland). The residue was dissolved with 5 mL of acetate buffer (pH 2.0) and stored in plastic specimen bottles in the freezer (- 4°C). 60 μL of each sample digest was dispensed into the cartridge of the analyzer and analyzed automatically for 76min. An integrator attached to the analyzer calculates the peak area proportional to the concentration of each of the amino acids.

### Physicochemical analysis of Soxhlet-extracted *C*. *mannii* oil

*a) Determination of lipid breakdown components*. Soxhlet-extracted *C*. *mannii* oil samples were analyzed for acid value (AV) as described by Arthur et al. [18] with slight modifications. About 1 mL of phenolphthalein solution (1%) was combined with 25 mL of ethanol before being properly neutralized with 0.1 M NaOH. A 15-s pink color was achieved by dissolving 1 g of oil in mixed neutral solvents and titration of the solution with 0.1 M NaOH. AV was expressed as mg KOH/g.

The oils were also analyzed for iodine (IV), peroxide (PV), and saponification (SV) as described by Badmus et al. [19] with slight modifications. For IV, 10 mL of carbon tetrachloride was used to dissolve the oil (5 g) in a 250 mL glass stopper flask and 20 mL of Hanus solution was added. After being mixed, the substance was left in the dark for 30 min. Using starch as an indicator, 15 mL of potassium iodide solution (10%) and 100 mL of water were added, stirred, and titrated with 0.1 M thiosulfate solution until a blue color developed just before the endpoint. The iodine value was calculated using the Eq 3 below:

$$IV=\frac{b-a\times N\times 1.269\times 100}{W}$$ 3

Where ‘a’ and ‘b’ are sample and blank titer values respectively, N is the normality of thiosulphate and W stands for the weight of the sample.

For PV, 5 g of the sample was mixed with 30 mL of a 3:2 combination of acetic acid and chloroform and swirled to dissolve the oil. 1 mL of potassium iodide solution was added to the mixture and occasionally stirred for 1 minute in the dark, and 30 mL of distilled water was added later. Using 1 mL of starch solution as an indicator, the solution was titrated with 0.01 N sodium thiosulphate solution. The titration process was vigorously shaken until the blue color completely vanished, showing that the chloromethane layer (CH_3_Cl) had been penetrated by the iodine molecule (I2). The PV had been calculated using the Eq 4 below:

$$PV=\frac{V\times N\times 100}{W}$$ 4

Where V is the volume of sodium thiosulfate, N is the normality of the titer, and W is the weight of the sample.

For SV, 1 g of oil and 25 mL of 0.1 M alcoholic potassium hydroxide was heated over reflux for 1 hour with constant shaking to complete saponification in an Erlenmeyer flask. Then, until a cloudy solution was created, 1 mL of phenolphthalein (1%) solution was added and titrated with the extra alkali with 0.5 M hydrochloric acid. The blank was run concurrently. The calculation for the SV was drawn up using the Eq 5 below:

$$SV=\frac{5.61\times (b-a)\times N}{W}$$ 5

where ‘a’ is the sample and ‘b’ is the blank titer value, N is 0.5 M normality of HCl, and W stands for the weight of 1 g oil.

Additionally, the oil samples were analyzed for thiobarbituric acid value (TBA)by the distillation method as described by Zen and Ullah [20] with slight modifications. Standard malondialdehyde (MDA) solution (1 mL) mixed with TBA (2 mL) subjected to a heated bath (95°C for 60 min), thereafter cooled and absorbance measured at 532 nm using Jenway electronic spectrophotometer (model no: 7305; England). Each sample (1 mL) was mixed with 2 mL TBA reagent. The TBARS was presented in mgMDA/kg.

*b) Determination of physical components*. The flash, and fire points, as well as the specific gravity of *C*. *mannii* oil samples, were analyzed as described by Onwuka [16]. For flash and fire points, a 10 mL volume of oil was poured into an evaporating dish and the temperature of the oil was gradually raised using an electric heater. A thermometer was suspended at the center of the dish ensuring that the bulb just dips inside the oil without touching the bottom of the dish. The temperature at which the oil began to flash when a flame was applied without supporting combustion was equally noted as the flash point, and the temperature at which the oil started to support combustion was recorded as the fire point. For specific gravity, a 50 mL pycnometer bottle was dried, weighed, filled with water, and weighed again. After drying, the bottle was filled with the oil sample and weighed. The weight of the oil obtained was divided by the weight of the water to obtain the specific gravity.
*c) Fatty acid profiling*. Soxhlet-extracted oil samples from *C*. *mannii* were analyzed for fatty acid composition, which employed a two-step methylation method that enabled the conversion to corresponding fatty acid methyl esters (FAMEs), as described by Badmus et al. [19]. *C*. *mannii* oil (40 μL) samples were mixed with 5.3 mL of methanol, and 0.7 mL of potassium hydroxide (10 M) solution, followed by reaction for 1.5 h at 55°C with intermittent mixing for 5 s at every 20 min. After cooling to room temperature, 580 μL of sulfuric acid (10 M) solution was added, and the reaction continued at 55° C for 1.5 h while being mixed for 5 sec every 20 min. Then, n-hexane (about 3 mL) was added and stirred for 5 min, thereafter the mixture was allowed to cool to room temperature, which was followed by centrifuging (5 min). Fatty acid profiling of extracts was performed using Agilent 6890 gas chromatograph equipped with a flame ionization detector (FID) and fused-silica capillary column (Omegawax; 30 m × 0.32 mm, id, film thickness 0.25 m, Supelco, Bellefonte, PA, USA). Nitrogen served as a carrier gas, which operated at a flow rate of 3.0 mL/min. The column temperature was programmed between 180 and 220°C (at a rate of 3°C/min), whereas the initial and final time runs were kept at 2 and 10 min, respectively. Additionally, the injector and detector temperatures were kept at 230 and 250°C, respectively. The sample volume was injected with a split ratio of 1:75. The FAMEs were identified by comparing their relative and absolute retention times with those obtained from gas chromatography—flame-ionization detection (GC-FID) FAME standards.

### Statistical analysis

All data that emerged from triplicate measurements were subject to analysis of variance (ANOVA), and subsequently represented by means ± standard deviation (SD). The level of statistical probability was set at p< 0.05 (95% confidence level). Mean separation was implemented by Fisher’s LSD (Least Significant Difference). Minitab^®^ 21.0 (Minitab, LLC, PA, USA) was used to run the data.

## Results and discussion

### Nutritional profile of (defatted and full fat) melon seed flour

Variations in the proximate composition of different melon seed flours are shown in Table 1. Defatted samples obtained significantly (*p<*0.05) higher proximate composition values, except the crude fat. Despite the defatted miniature sample’s higher moisture (11.91%), the crude ash, fat, and fiber range for large seed flour and miniature melon seed flour seemed comparable. The defatted large melon seed obtained the maximum carbohydrate content (16.26%), followed by the defatted miniature melon seed (12.36%). Overall, the approximate composition in this document competes well with previously reported data [2, 7, 9, 21–23]. Various proximate trends have been previously reported especially for resembling defatted flour samples. Whereas Ogunbusola *et al*., [2] and Omowaye-Taiwo [21] observed lower moisture, Ogunbusola *et al*., [2], Omowaye-Taiwo *et al*., [21], Edith *et al*. [22] and Akusu *et al*., [23] observed higher fat contents. In addition, the protein contents of *C*. *mannii* here seem higher than those of other *Curcubitacea* seeds [9, 24].

**Table 1:** Variations in proximate composition of different melon seed flours (g/100g).

samples	Moisture	Ash	Fat	Protein	Crude fiber	Carbohydrate
Full-fat large, *C*. *mannii*	6.93^d^±0.01	2.78^c^±0.01	49.83^a^±0.01	35.46^d^±0.00	1.57^c^±0.01	3.43^c^±0.03
Full-fat miniature *C*. *mannii*	7.85^c^±0.01	2.39^d^±0.00	48.34^b^±0.01	37.45^c^±0.00	1.33^d^±0.01	2.63^d^±0.03
Defatted Large *C*. *mannii*	10.86^b^±0.00	3.89^a^±0.01	0.24^c^±0.00	66.44^b^±0.18	2.32^b^±0.01	16.26^a^±0.19
Defatted miniature *C*. *mannii*	11.91^a^±0.01	3.78^b^±0.06	0.23^c^±0.00	69.04^a^±0.01	2.68^a^±0.01	12.36^b^±0.07

Values are mean ± standard deviation of triplicate determinations. Means in the same column with different superscripts ^a-d^ are significantly different (p<0.05)

Variations in the mineral composition of different melon seed flours are shown in Table 2. The different melon seed flours demonstrated enriched mineral compositions, from calcium (Ca), magnesium (Mg), phosphorus (P), sodium (Na), potassium (K), iron (Fe), zinc (Zn), to manganese (Mn). The defatting process would significantly improve (*p<*0.05)the mineral content of the flour samples. Na (ranging from 223.92–246.99 mg/100g) and K (ranging from 211.04–178.45 mg/100g) appear to be the most abundant, while P (ranging from 1.78–1.92 mg/100g) and Mn (ranging from 1.14 to 1.64 mg / 100g) appear least abundant in the melon seed flour of this study, corroborating the data reported elsewhere [3]. The function of P is for bone mineralization and energy transfer, Ca for bone development and growth, Mg for cardiac and smooth muscle contractibility, K for contraction of smooth, skeletal, and cardiac muscle, as well as Na for nerve transmission and regulation of fluid balance [25]. Mn is a cofactor for enzymes involved in carbohydrate metabolism, Fe synthesizes hemoglobin and myoglobin, and Zn serves as an antioxidant defense function and synthesis of DNA/RNA [26].

**Table 2:** Variations in mineral composition (mg/100g) of different melon seed flours.

Sample	Ca	Mg	P	Na	K	Fe		Zn	Mn
Full-fat large *C*. *mannii*	53.88^c^±0.01	61.24^d^±0.01	1.78^c^±0.01	231.44^c^±0.01	195.75^c^±0.01	2.26^d^±0.01		2.86^c^±0.01	1.56^b^±0.01
Full-fat miniature *C*. *mannii*	49.86^d^±0.01	64.33^c^±0.01	1.88^ab^±0.00	223.92^d^±0.01	178.45^d^±0.01	2.37^c^±0.00		2.65^d^±0.01	1.64^a^±0.01
Defatted large *C*. *mannii*	58.92^a^±0.01	72.87^b^±0.01	1.86^b^±0.01	246.99^a^±0.01	211.04^a^±0.00	2.69^b^±0.01		3.15^b^±0.01	1.30^c^±0.00
Defatted miniature *C*. *mannii*	57.63^b^±0.00	75.09^a^±0.01	1.92^a^±0.01	236.37^b^±0.01	207.54^b^±0.01	2.86^a^±0.01	3.66^a^±0.01		1.14^d^±0.01

Values are mean ± standard deviation of triplicate determinations. Means in the same column with different superscripts ^a-d^ are significantly different (p<0.05)

Variations in vitamin concentrations of different melon seed flours are shown in Table 3. Significant differences (*p<*0.05) in vitamin concentrations occurred between samples with a peak Vit. B_1_ (0.712 μg/100g) in the defatted miniature melon seed flour, and the least vitamin B_3_ (0.226 mg / 100g) in the full-fat large melon seed flour. However, a high carotenoid (0.678 μg/100g in miniature seed being the highest) in full-fat samples depicts a more rapid conversion to retinol (vitamin A) compared to defatted melon seed flour samples. As health supplements capable of preventing cancer, carotenoids should protect cells against oxidative damage, thus acting as an antioxidant agent [27]. Also, the thiamine (Vit B_1_) data herein seems higher than the recommended daily intake of 0.2 to 1.0 mg for less than infants of one year of age [9].

**Table 3:** Variations in vitamin concentration of different melon seed flours.

Sample	Carotenoid (μg/100g)	Vitamin B_1_ (mg/100g)	Vitamin B_2_ (mg/100g)	Vitamin B_3_ (mg/100g)
Full-fat large *C*. *mannii*	0.678^a^±0.00	0.367^d^±0.00	0.453^d^±0.01	0.226^c^±0.01
Full fat miniature *C*. *mannii*	0.664^b^±0.00	0.476^c^±0.01	0.535^c^±0.01	0.235^bc^±0.00
Defatted large *C*. *mannii*	0.352^d^±0.01	0.552^b^±0.00	0.678^b^±0.01	0.241^b^±0.01
Defatted miniature *C*. *mannii*	0.571^c^±0.01	0.679^a^±0.01	0.712^a^±0.01	0.265^a^±0.00

Values are mean ± standard deviation of triplicate determinations. Means in the same column with different superscripts ^a-d^ are significantly different (p<0.05)

Variations in functional properties of different melon seed flours are shown in Table 4. Significant differences (*p<*0.05) in functional properties when comparing full-fat and defatted melon seed flours. Specifically, defatted melon seed flours obtained higher OAC, WAC, SI, and FC, whereas full fat obtained higher BD and EC. A high bulk density would be desirable to reduce paste thickness, which eases the dispersibility of food powders [21]. This situation might account for the efficacy of raw melon seed flour in soup making. Differences in swelling indices might be suggesting the intermolecular binding nature of starch within the raw melon seed. The high OAC and WAC of the defatted melon seeds may also be suggesting, either, the high hydrophobic protein of the seeds, or the accessibility of water-binding sites on side-chain groups of proteins previously blocked in a lipophilic environment, respectively [21]. The OAC of full-fat flour samples fell below the data recorded by Ogunbusola *et al*. [2], Omowaye-Taiwo *et al*. [21], Akusu [23], and Thierry *et al*. [28]. Overall, the WAC data here compete with full-fat /defatted seed flours reported elsewhere [3, 21, 23, 28].

**Table 4:** Variations in functional properties of different melon seed flours.

Sample	BD (g/ml)	OAC (g/g)	WAC (g/g)	SI (g/ml)	FC (%)	EC (%)
Full-fat large *C*. *mannii* flour	1.833^a^±0.00	0.800^b^±0.00	0.667^b^±0.06	2.217^c^±0.01	35.87^d^±0.01	22.68^c^±0.00
Full-fat miniature C. *mannii* flour	1.171^b^±0.01	0.767^b^±0.06	0.667^b^±0.12	2.218^c^±0.01	41.78^c^±0.01	23.44^a^±0.01
Defatted large *C*. *mannii* flour seed flour	0.932^c^±0.01	1.667^a^±0.06	1.567^a^±0.06	5.217^b^±0.01	53.78^b^±0.18	22.35^d^±0.00
Defatted miniature *C*. *mannii* seed flour	0.773^d^±0.00	1.733^a^±0.06	1.633^a^±0.06	5.749^a^±0.02	55.24^a^±0.01	23.18^b^±0.01

Values are mean ± standard deviation of triplicate determination. Means in the same column with different superscripts ^a-d^ are significantly different (p<0.05).

The amino acid profile of melon seeds flours is shown in Table 5, which showed significant difference (*p<*0.05) between both samples. The miniature *C*. *mannii* seeds were richer in most essential and non-essential amino acids compared to the large ones. When comparing the essential amino acid content of melon seeds with the reference standard [29], *C*. *mannii* seeds possess some quantity of leucine, methionine, tyrosine, phenyl, and alanine and valine, with somewhat moderate histidine, valine, and tryptophan. This situation would suggest the amino acid distributions in the melon seed as rather higher in the miniature melon seeds compared to the large seeds of this study. Notably, Besong [30] equally reported *C*. *mannii* with reduced essential amino acids. It is important to reiterate that amino acids are broadly classified as essential and nonessential types [31]. Moreover, this current study clearly show that *C*. *mannii* possesses essential amino acids, which should help meet the protein demands of children and adults in both developed and developing countries.

**Table 5:** Amino acid profile of different defatted melon seeds flours.

Amino acid (g/100g)	Miniature melon seeds	Large melon seeds
Threonine	2,17 ^a^ ± 0,09	1,83 ^a^ ± 0,04
Leucine	5,11 ^a^ ± 0,16	4,31 ^a^ ± 0,44
Isoleucine	1,72 ^a^ ± 0,11	0,77 ^b^ ± 0,05
Lysine	2,15 ^a^ ± 0,01	1,29 ^b^ ± 0,02
Methionine	3,06 ^a^ ± 0,06	2,46 ^b^ ± 0.05
Phenylalanine	2,76 ^a^ ± 0,12	1,80 ^b^ ± 0.13
Tyrosine	0,77 ^a^ ± 0,03	0,47 ^b^ ± 0.03
Valine	0,98 ^a^ ± 0,01	0,60 ^b^ ± 0.01
Arginine	0,26 ^a^ ± 0,03	0,15 ^b^ ± 0.01
Histidine	0,19 ^a^ ± 0,00	0,19 ^a^ ± 0.00
Alanine	0,57 ^a^ ± 0,01	0,25 ^b^ ± 0.05
Aspartic acid	1,29 ^a^ ± 0,01	1,32 ^a^ ± 0.01
Asparagine	1,19 ^a^ ± 0,01	0,74 ^a^ ± 0.02
Glutamic acid	2,54 ^a^ ± 0,04	2,48 ^a^ ± 0.01
Glutamine	2,50 ^a^ ± 0,01	1,17 ^b^ ± 0.01
Glycine	0,67 ^a^ ± 0,02	0,44 ^b^ ± 0.01
Proline	0,87 ^a^ ± 0,03	1,06 ^a^ ± 0.06
Serine	0,22 ^a^ ± 0,01	0,17 ^b^ ± 0,01
Tryptophan	0,20 ^a^ ± 0,01	0,14 ^a^ ± 0.02
Cystine	1,19 ^a^ ± 0,01	0,79 ^b^ ± 0,01
Total amino acids	30,36 ^a^ ± 0,25	22,36 ^b^ ± 0,01

Values are mean ± standard deviation of triplicate determination. Means in the same row with different superscripts ^a-b^ are significantly different (p<0.05)

### Physicochemical aspects of different Soxhlet extracted melon seed oils

Lipid breakdown and associated physical aspects of different Soxhlet-extracted melon seed oils are shown in Table 6. The TBA, AV, and PV seemed low, whereas the IV and SV seemed high. The reduced TBA value of both *C*. *mannii* oils (0.030 mg/kg large) and (0.038 mg/kg miniature) suggests limited lipid peroxidation with storage. The PVs were statistically different (*p<*0.05) between the two samples, with 2.95 mEqO_2_/kg large seed oil and 3.94 mEqO_2_/kg miniature seed oil, both lower than another melon seed oil (8.3 mEqO_2_/kg) reported by [32]. The IV, which helps to ascertain the quality of *C*. *mannii* seed oils was quite high (large seed = 122.40 g I_2_/100g; miniature seed = 116.32 g I_2_/100g), suggesting a high degree of fatty acid unsaturation. Elsewhere, varying IVs have been reported, like 132.6 g I_2_ /100g for *C*. *mannii* [7], 108.33 g / 100 g for watermelon, 104.45 mg /100g for pumpkin oil [33], as well a range from 15.10 to 45.80 in five varieties of melon [34]. This specific acid value suggests the edibility of oil and its food processing suitability [19]. The *C*. *mannii* oils appear with low acid values < 2 mg KOH/g, which is below the Codex standard value of virgin vegetable oil. This would suggest that *C*. *mannii* oil will not require pretreatment before transesterification for biodiesel production [7, 24]. Interestingly, both high flash and fire points of the different Soxhlet-extracted oils significantly differed (*p<*0.05) but not so for specific gravity (*p>*0.05). Typically, both flash and fire points are crucial factors employed in ascertaining the lubricant capacity of any given oil. For emphasis, the flash point refers to the lowest temperature (under the conditions of test laboratories) at which the vapor of lubricant could start to catch fire/ignite when exposed to a naked fire/spark. However, after flash point temperature, if the heating of lubricant is persistently increased, the kinetic energy of lubricant would rapidly ease the release of vapor to produce a fire for a minimum of 5 s, and the temperature at which this happens is called fire point. It is widely understood that every flash point temperature is lower than that of a fire point [35].

**Table 6:** Lipid breakdown and associated physical aspects of different Soxhlet-extracted melon seed oils.

Properties	Large C. *mannii*	Miniature C. *mannii*	p-Value	Level of statistical significance (p<0.05)
**Flashpoint (unit)**	352.00±2.65	337.33±2.08	0.002**	Significant
**Fire point (unit)**	358.67±1.16	350.67±1.16	0.001**	Highly significant
**TBA (mg MDA/kg)**	0.030±0.00	0.038±0.01	0.168	Not significant
**Iodine value (g I**_**2**_ **/100g Oil)**	122.40±0.73	116.32±0.45	0.000**	Highly significant
**Acid value (mg KOH/g)**	1.08±0.01	1.27±0.07	0.007**	Highly significant
**Peroxide value (**mEqO_2_/kg**)**	2.95±0.01	3.94±0.04	0.000**	Highly significant
**Saponification value (mg KOH/g)**	188.09±1.03	183.92±6.34	0.323	Not significant
**Specific gravity (kg/dm** ^**3**^ **)**	0.99±0.00	0.96±0.02	0.132	Not significant

Values are mean ± standard deviation of triplicate determination. Means in the same row with different superscripts ^a-b^ are significantly different (p<0.05)

The fatty acid profiles of different oils of melon seed oils are shown in Table 7. A total of twenty fatty acids were detected. For the monounsaturated fatty acids (MUFA), the oleic acid appears strong in both seed oils. The large seed oil had higher oleic acid (3.963%) compared to the miniature (2.629%) type, wherein both showing a significant difference (*p<*0.05). For polyunsaturated fatty acids (PUFA), both linoleic and linolenic essential fatty acids were the main components, wherein linoleic appeared more concentrated than linolenic, This was in agreement with previously reported data of melon seed oil [36, 37]. The high content of linoleic acid and linolenic acid provides this oil an important nutritional status [38]. Although an omega-3-fatty acid with good health effects, linolenic acid is easily susceptible to peroxidation that leads to off-flavors and harmful oxidation products. Notably, Warner and Gupta [32] reported that decreased (from 2 to 0.8%) linolenic acid content in oils depicted improved flavor quality and oxidative stability especially in fried food. It is believed that the consumption of MUFA and PUFAs could lower the total plasma cholesterol and low-density lipoprotein cholesterol (LDL) in human subjects [30]. Moreover, unsaturated fats when consumed are nutritionally desirable and may lower the blood cholesterol levels [7]. Indeed, palmitoleic and stearic acids remain among the saturated fatty acids (SFA) in the melon seed oils, like those found in sunflower, wheat germ, and pumpkin seed [39, 40], much less in those found in palm (where oleic acid is most abundant) and coconut oils (that have mostly saturated fatty acids, palmitic and lauric acids) [41]. Moreover, typical seed oil has always been poised as highly polyunsaturated, with about 63% linoleic acid and 16% oleic acid, and serve as an enriched source of vitamins C and B2, minerals, riboflavin, fats, and carbohydrate [4, 9].

**Table 7:** Fatty acid profiles of different Soxhlet-extracted melon seed oils.

Fatty acid profile components	Miniature *C*. *mannii* oil extract (mg/mL)	Large *C*. *mannii* oil extract (mg/mL)
Linoleic	5,00 ^b^ ± 0,00	6,45 ^a^ ± 0,08
Arachidonic	1,57 ^b^ ± 0,05	2,93 ^a^ ± 0,02
Oleic	2,59 ^b^ ± 0,06	3,91 ^a^ ± 0,07
Ricinoleic	4,91 ^b^ ± 0,12	6,27 ^a^ ± 0,01
Palmitoleic	0,61 ^b^ ± 0,02	0,93 ^a^ ± 0,03
Stearic	0,81 ^b^ ± 0,02	1,96 ^a^ ± 0,04
Linolenic	0,19 ^a^ ± 0,00	0,26 ^a^ ± 0,04
Caproic	1,39 ^b^ ± 0,01	1,94 ^a^ ± 0,02
Myristic	1,27 ^b^ ± 0,01	2,24 ^a^ ± 0,04
Margaroleic	3,82 ^a^ ± 0,09	4,16 ^a^ ± 0,07
Petroselenic	1,93 ^b^ ± 0,05	2,90 ^a^ ± 0,02
Vaccenic	0,83 ^a^ ± 0,07	0,93 ^a^ ± 0,05
Behenic	3,29 ^a^ ± 0,37	4,10 ^a^ ± 0,02
Cetoleic	0,26 ^b^ ± 0,01	0,58 ^a^ ± 0,05
Erucic	1,35 ^b^ ± 0,06	1,91 ^a^ ± 0,05
Nervonic	1,75 ^b^ ± 0,06	2,69 ^a^ ± 0,08
Margaric	5,78 ^b^ ± 0,13	6,17 ^a^ ± 0,10
Lauric	3,63 ^a^ ± 0,07	5,82 ^a^ ± 0,09
Capric	0,96 ^b^ ± 0,03	1,73 ^a^ ± 0,01
Butyric	1,30 ^b^ ± 0,11	1,97 ^a^ ± 0,03
Total Fatty acids	43,22 ^b^ ± 1,08	59,86 ^a^ ± 0,55

Values are mean ± standard deviation of triplicate determination. Means in the same row with different superscripts ^a-b^ are significantly different (p<0.05)

### Storage performance of different Soxhlet-extracted melon seed oils

Understanding the storage performance of crop/food oils is essential as it demonstrates the product’s commercial potential, as it would be underpinned by outcomes of measurable quality attributes, especially those that show the degree of oil degradability. In the current work, the storage performance of the different Soxhlet-extracted melon seed oils was determined by way of peroxide (PV) and thiobarbituric acid (TBA) values, as shown in Table 8. Concerning the peroxide value, significant increases were found with storage time (*p>*0.05), which did not exceed the Prevention of Food Adulteration Act (PFA) limits for fresh oils (10 mEqO_2_/kg) [42]. The slight sl increase in peroxide values could have happened because of the higher activity of lipoxygenase enzymes facilitated by elevated temperatures. However, it would suggest that the oils may not be easily susceptible to oxidative rancidity. More so, factors influencing peroxide values can include the state of oxidation (quantity of oxygen consumed), as well as the breakdown of the primary oxidation products like hydroperoxides into secondary oxidation products [43]. Concerning the TBA values, some resemblances appeared within the first four months (*p>*0.05), which became different by month 6 given the noticeable increases (*p<*0.05). Such increases in TBA values would likely suggest the onset of off-flavor development, which might not be sufficient to bring about observable/perceptible changes. Additionally, the overall TBA data were all below 1.00 mg MDA/kg value, which would suggest the somewhat promising stability of the melon seed oil in this study. It is also possible that the high amount of oleic acid, combined with the lesser amount of linoleic acid might be contributing to this overall low TBA data. It should also be noted that TBA value is among the important parameters that detect the deterioration of seed oil [44].

**Table 8:** Peroxide and thiobarbituric acid value of different Soxhlet-extracted melon seed oils with storage time.

Period (Month)	Peroxide value (mEqO_2_/kg)			Thiobarbituric acid value (mg MDA/kg)		
	Large *C*. *mannii* oil extract	Miniature *C mannii* oil extract	p-Value	Large *C*. *mannii*	Miniature *C*. *mannii*	p-Value
0	1.95^d^ ±0.02	2.94^d^ ±0.04	0.000**	0.030^d NS^±0.00	0.038^d NS^±0.00	0.168
2	3.32^c^±0.02	4.12^c^ ±0.02	0.000**	0.088^c NS^±0.00	0.096^c NS^±0.00	0.088
4	5.64^b^ ±0.04	4.34^b^ ±0.00	0.000**	0.163^b NS^±0.00	0.163^b NS^±0.00	0.947
6	7.98^a^± 0.01	8.55^a^ ±0.02	0.000**	0.287^a^ ±0.01	0.257^a^ ±0.01	0.009**

Values are mean ± standard deviation of triplicate determinations. Means in the same column with different superscripts ^a-d^ are significantly different (p<0.05), while **: Means in the same row are significantly (p<0.05) different. NS: Means in the same row are not significantly (p>0.05) different.

## Conclusions

Nutritional profile of defatted and full-fat flour, alongside physicochemical properties and storage performance of Soxhlet-extracted oil of white melon (*C*. *mannii*) seed varieties from Southeast Nigeria were investigated. Nutrient composition and functional properties of the flour make the studied *C*. *mannii* seed varieties a very promising crop in food processing. The acid, iodine, peroxide, and saponification values of *C*. *mannii* seed oils at both varieties fell within the recommended limits, which suggested less susceptibility to spoilage. The high linoleic acid content and low linolenic acid content of *C*. *mannii* seed oil poised it as a reliable resource for cooking, and probably, a potential antidote against cardiovascular disease. Moreover, the *C*. *mannii* seeds given their underutilized nature can serve as good substitutes in food formulations to reduce the pressure on groundnuts, soya beans, and other related seeds commonly used for food formulations and oil extraction. The direction of future studies should be on stability of the oils using different storage conditions. Such future study should help show which storage condition(s) would best suit this Soxhlet-extracted *C*. *mannii* seed oil. Future work also needs to identify the level of anti-nutritional factors as well as nutrient bioavailability in *C*. *mannii* seeds, in order to justify their usage, either as animal feed, for human consumption, or as an industrial oil feedstock. Another future work should be to identify secondary metabolites in *C*. *mannii* seeds that could emerge as potential bioactive compounds/nutraceuticals for disease prevention/management.
